# Uncovering the evolving drivers of student engagement in physical education: a dynamic analysis across developmental stages

**DOI:** 10.3389/fpsyg.2025.1721921

**Published:** 2026-01-08

**Authors:** Zeren Lv

**Affiliations:** Luoyang Normal University, Luoyang, China

**Keywords:** dynamic systems, MICMAC, physical education, time-varying influences, transient dominance

## Abstract

**Purpose:**

To address the limitations of static and subjective methods, this study aims to examine the dynamic interplay among factors influencing student engagement in physical education across different developmental stages.

**Method:**

This study integrates an enhanced Delphi method with a Time-Varying Influence Network model and dynamic MICMAC analysis to compute time-varying driver power and dependency from the evolving total influence matrix.

**Results:**

The dynamic system evolution was characterized by three key patterns: convergence toward equilibrium, transient dominance, and structural stability.

**Discussion/conclusion:**

The dynamic system evolution is characterized by three key patterns: convergence toward equilibrium, transient dominance, and structural stability. These results underscore the importance of timing and targeting in intervention strategies and the need to reinforce stable core elements.

## Introduction

At different stages of a school’s development, the status of various factors related to student engagement undergoes dynamic changes, and the extent to which these factors promote engagement also shifts accordingly. In this study, the term “developmental stages” refers to a school’s progression at the organizational level—a composite construct capturing its holistic condition through the distinct and evolving configuration of core elements such as administrative practices, student cohort composition, curriculum systems, and teaching staff allocation. Given the practical challenge of obtaining an accurate and consistent assessment of each individual factor’s state, examining the interactions among them yields greater practical insight. Since enhancing student engagement in physical education remains a primary focus in educational practice, this study emphasizes analyzing the interplay among influencing factors across these organizational phases. By adopting this holistic lens and exploring how inter-factor relationships evolve over time, we aim to provide a more scientifically grounded basis for guiding the design and implementation of teaching activities.

To better understand the factors influencing student engagement, expert prediction methods such as the Delphi method have been widely used to assess the importance of factors and assign their weights. However, the traditional Delphi method exhibits significant subjectivity in the weighting process, relying heavily on experts’ empirical judgments or simple averaging, which can compromise the consistency and objectivity of the results (Rowe and Wright, 2011).

To address this limitation, the present study introduces a hybrid approach that integrates Bootstrap resampling with ridge regression. Bootstrap enhances objectivity by generating multiple expert subsamples, thereby robustly estimating the weighting structure (Nevitt and Hancock, 2001), while ridge regression mitigates multicollinearity among expert ratings and improves weight stability through regularization (McDonald, 2009). Together, these methods systematically reduce reliance on subjective judgment, offering a more statistically grounded and replicable framework for deriving influence weights—thus directly addressing the subjectivity inherent in traditional Delphi applications.

Meanwhile, MICMAC analysis, as an important tool for identifying the degree of mutual influence among factors within a system, shows unique advantages in revealing the structural characteristics of complex systems. However, its traditional static nature cannot reflect dynamic influence processes over time, which limits its depth and breadth in practice (Jiang et al., 2019). This restriction is particularly salient in dynamic contexts such as educational engagement, where driver prominence and dependency shift across developmental stages.

To overcome this static barrier, this study integrates a Time-Varying Influence Network (TVIN) model with MICMAC, enabling dynamic MICMAC analysis. The TVIN model employs differential equations and a state-feedback mechanism to continuously update the influence matrix based on node states over time, thereby simulating system evolution from initial to steady-state conditions. This integration allows for the temporal tracking of driving and dependence powers across multiple stages (Clemen and Winkler, 1999; Jetter and Schweinfort, 2011). By transforming MICMAC from a static structural tool into a time-sensitive analytical framework, this approach addresses the critical gap in temporal dynamics analysis of engagement drivers, offering a more nuanced understanding of how and when different factors exert influence in physical education settings.

A critical yet overlooked dimension in understanding student engagement is its temporal evolution. Static analyses fail to capture how drivers shift in influence across developmental stages, limiting intervention strategies to generalized approaches. By integrating a Time-Varying Influence Network (TVIN) with dynamic MICMAC analysis, this study introduces a temporally sensitive framework that traces how driving and dependence powers change over time. This allows for the identification of stage-specific dominant factors, reveals transient versus stable influences, and supports the design of phase-adaptive interventions. Thus, our approach directly addresses the gap in temporal analysis of engagement drivers, offering a dynamic perspective essential for timely and targeted educational strategies.

In higher education, physical education courses play a vital role not only in promoting students’ physical and mental health, but also in fostering teamwork and competitive awareness. Research indicates that physical activity significantly enhances students’ physical fitness, psychological well-being, and social skills. However, student participation in such courses remains low due to multifaceted reasons, including lack of interest, misalignment between course content and student needs, and inadequate assessment mechanisms (Keegan et al., 2019; Fairclough and Stratton, 2006; Babic et al., 2014; Caspersen et al., 1985). Students’ perceptions and attitudes further influence engagement, with many viewing physical education merely as a graduation requirement rather than as integral to personal development.

Although interventions such as innovative teaching methods, curriculum redesign, and expanded extracurricular opportunities have been proposed to increase student activity levels, their implementation faces considerable challenges. Limited institutional resources and variability in teachers’ professional competence often hinder full execution and effectiveness. Moreover, as noted by Lonsdale et al. (2013), enhancing students’ exercise intensity and participation is a complex, multidimensional issue that extends beyond pedagogical adjustments. It requires attention to individual differences, socio-environmental factors, and policy support.

Thus, effectively improving college students’ engagement in physical education demands comprehensive strategies that are adaptable to evolving student needs and social contexts. Ongoing efforts should also aim to educate students on the intrinsic value of physical education and cultivate positive attitudes toward sustained participation (Spittle et al., 2009; Xiang et al., 2017).

Self-Determination Theory (SDT) offers a theoretical framework for understanding student motivation, positing that the fulfillment of three basic psychological needs—autonomy, competence, and relatedness—is fundamental (Ryan and Deci, 2017). In education, teacher behaviors that support these needs are pivotal for cultivating autonomous motivation, thereby enhancing student engagement and learning (Reeve and Cheon, 2021; Ahmadi et al., 2023). Recent work to systematize SDT-based interventions has produced a classification system of teacher motivational behaviors (TMBs), which specifies concrete actions that either support or thwart these psychological needs (Ahmadi et al., 2023). Grounded in this theoretical and empirical foundation, the present study investigates the dynamic interplay of factors influencing student engagement in physical education, examining how these factors map onto SDT’s core constructs across developmental stages.

To address these limitations, this study proposes an integrated methodological framework consisting of three key components. First, we integrate bootstrapping resampling with ridge regression to refine the weighting of expert scores. This approach mitigates multicollinearity among variables and enhances the objectivity of weight assignment. Specifically, the regularization property of ridge regression reduces estimation bias and improves weight reliability, particularly in small-sample contexts with correlated predictors, thereby overcoming the subjectivity and inconsistency inherent in traditional Delphi methods. Furthermore, bootstrapping generates multiple subsamples, strengthening the robustness of statistical inferences.

Second, we develop a Time-Varying Influence Network (TVIN) model to dynamically trace the evolution of direct influence matrices among key factors (Chai et al., 2021). Incorporating a state feedback mechanism, the TVIN model captures the temporal dependencies and evolving interaction patterns within complex systems over time (Haider and Levis, 2008).

Finally, we integrate the TVIN model with dynamic MICMAC analysis, enabling a time-varying examination of the driving and dependent forces behind student engagement. This combined approach not only elucidates the dynamic roles of various factors but also informs the adaptation of intervention strategies across different temporal phases in response to shifting environmental and social conditions.

Collectively, this innovative framework advances the scientific rigor of expert weight calibration and offers novel analytical perspectives for uncovering the internal dynamics of educational systems. We anticipate that these methodological contributions will support the enhancement of teaching effectiveness in university physical education programs.

## Method

### The Delphi method

The Delphi Method is a method that serves as the core of expert forecasting, aiming to gradually reach a consensus on a complex issue or future trend by organizing multiple rounds of expert opinion solicitation and feedback. The method is often used in areas where there is a lack of sufficient historical data or a high degree of uncertainty. The core process involves first selecting a group of experts with rich knowledge and experience in the relevant field, and then collecting their opinions and predictions on a specific issue through an anonymous questionnaire.

Based on the established methodological framework, a Delphi study was conducted to identify and validate the key factors influencing student engagement in PE. The procedure comprised two sequential phases: (1) the identification and prioritization of core factors, and (2) the assessment of their pairwise influence relationships.

### Expert panel recruitment

An expert panel of 12 members was assembled via purposive sampling based on three criteria: (1) diverse professional backgrounds (frontline PE teachers, teaching administrators, and academic researchers in sports pedagogy); (2) extensive professional experience (minimum 10 years); and (3) diverse institutional affiliations across various universities and educational management units. The panel was gender-balanced (7 male, 5 female) and included five experts holding doctoral degrees and four with master’s degrees (see Table 1 for full demographics).

**Table 1:** Basic information of experts.

Name	Gender	Age	Education	Work year	Access mode
Teacher A	Male	52	BA	29	On-site
Teacher B	Male	59	BA	38	On-site
Teacher C	Female	46	MA	23	On-site
Teacher D	Female	38	BA	26	On-site
Teaching Manager A	Male	37	MA	10	On-site
Teaching Manager B	Male	43	MA	16	Telephone interview
Teaching Manager C	Female	42	PHD	12	On-site
Teaching Manager D	Female	55	PHD	24	Telephone interview
Professor A	Male	50	PHD	19	Telephone interview
Professor B	Male	58	MA	32	Telephone interview
Professor C	Female	55	PHD	23	On-site
Professor D	Female	54	PHD	21	Telephone interview

#### Phase 1: identification and prioritization of core factors

The first phase aimed to generate and reach consensus on a set of core factors. It began with an open-ended round where experts listed potential factors affecting student engagement, yielding an initial pool of 28 items. These items were synthesized by merging overlaps and removing out-of-scope suggestions, then categorized into four dimensions (Teacher, Student, Course, Management), resulting in 18 candidate factors.

Subsequently, three rating rounds were conducted. Experts rated the importance of each factor on a 5-point Likert scale (1 = not important to 5 = extremely important) and provided qualitative comments. After each round, personalized feedback reports—containing the group’s mean, standard deviation, coefficient of variation (CV), interquartile range (IQR), and anonymized comments—were returned. Experts were asked to reconsider their ratings in light of this feedback.

Consensus was defined *a priori* as a CV < 0.25, an IQR ≤ 1, and a mean importance rating ≥ 3.5. This process concluded after the fourth round (including the initial open round), with 14 factors meeting all consensus criteria. These factors constituted the final set for dynamic analysis.

#### Phase 2: Assessment of pairwise influence relationships

A second, independent Delphi process was conducted to evaluate the direct influence between the 14 identified factors. The same panel and consensus criteria (CV < 0.25, IQR ≤ 1, mean ≥ 3.5) were applied across four rounds.

Experts were instructed to assume all factors were standardized to a common scale of impact on engagement, allowing them to focus solely on the relative strength of direct causal links. They rated the influence of factor *j* on factor *i* using a 14 × 14 matrix and a 6-point scale: 0 (none), 1 (very weak), 2 (weak), 3 (moderate), 4 (strong), 5 (very strong).

Following each round, personalized feedback—containing the group’s statistical summary and anonymized comments—was provided. Experts revised their ratings in subsequent rounds based on this aggregated information. The process concluded after the fourth round when consensus was achieved for all rated relationships, yielding a stable direct influence matrix from each expert.

**Figure 1:**
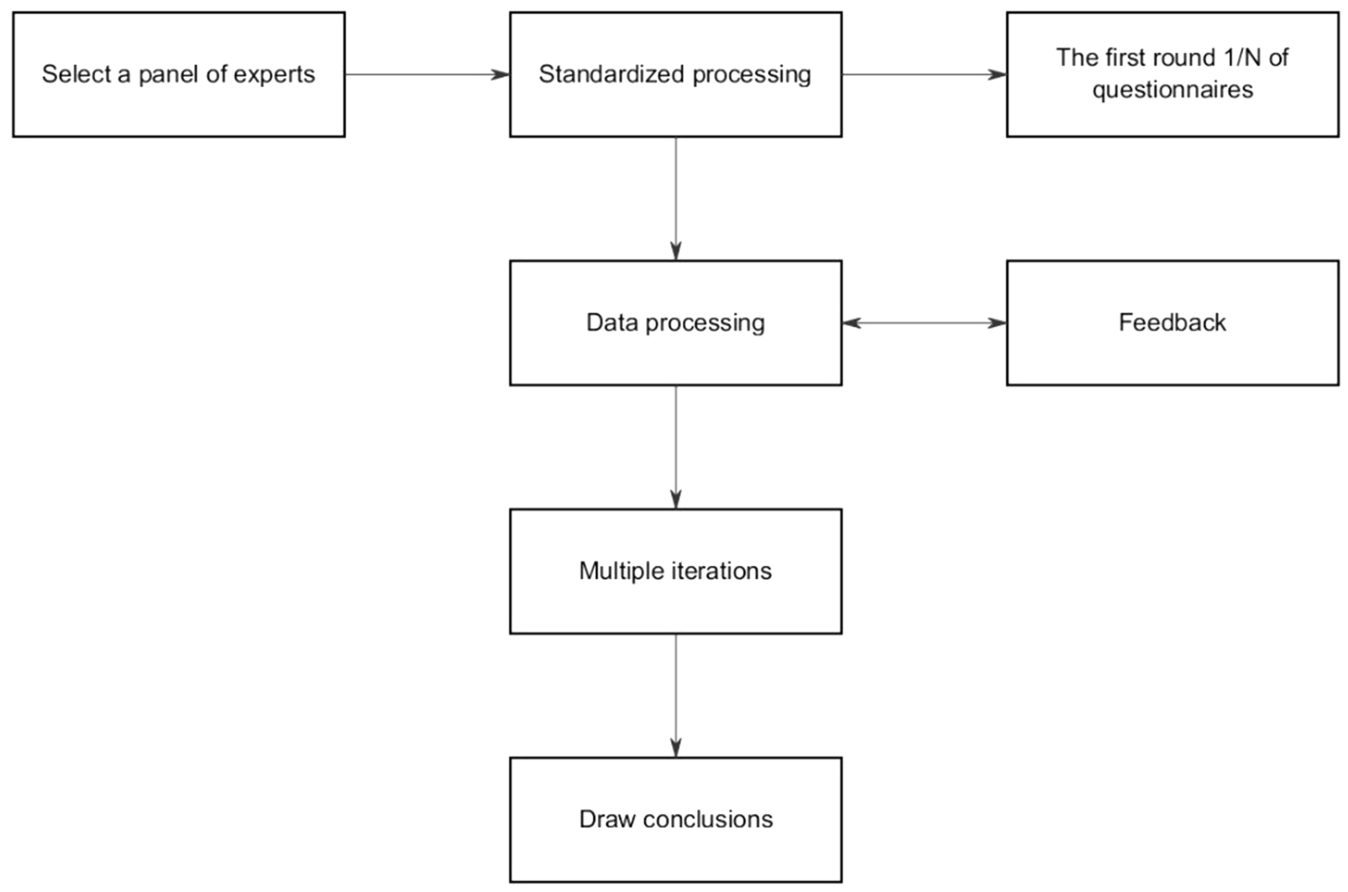
The technical route of the Delphi method.

Its technical route is shown in Figure 1.

In this study, 12 experts and scholars were invited to participate in the Delphi method, and the information of the experts is shown in Table 1.

After several rounds of opinion solicitation and feedback, and after systematic screening and integration, 14 core factors with significant influence on participation were finally identified as shown in Table 2. In order to ensure the comparability and consistency of the assessment results, during the expert review process, all influencing factors were standardized with respect to their potential to positively promote student engagement.

**Table 2:** Influencing factor definition.

Dimension	Factors	Definition
Teacher	Professional teaching ability (A1)	Professional teaching ability refers to a series of professional knowledge, skills and abilities that teachers show in teaching activities in specific subject areas.
	Teaching methods (A2)	Teaching methods are a general term for the methods and means used by teachers and students in the teaching process in order to achieve common teaching goals and complete common teaching tasks.
	Teaching work attitude (A3)	Teaching work attitude refers to the professional attitude and psychological state shown by teachers in teaching activities.
Student	Learning Methods (A4)	Learning methods are formed during the learning process, which conforms to personal characteristics and can improve learning efficiency.
	Student course adaptation (A5)	Student curriculum adaptation refers to the coordination of students’ learning behavior and learning psychological qualities with social structure and social interaction process.
	Student course identification (A6)	Student curriculum recognition refers to the degree of recognition and acceptance of the value, goals, content, teaching methods and other aspects of the courses they have learned.
	Personal preferences (A7)	Personal preference refers to the tendency and preferences that an individual shows for a certain option when facing different choices.
Course	Interesting course (A8)	Classroom fun refers to the fact that in the teaching process, through various methods and means, the classroom atmosphere is more active, so that students can learn in a relaxed and pleasant environment, thereby improving their interest and efficiency in learning.
	Course interactive design (A9)	Curriculum interactive design refers to a teaching method in curriculum design that enhances students’ interest in learning, promotes internalization of knowledge, and cultivates comprehensive abilities through creating links.
	Course practice session (A10)	The course practice link refers to the course in which students can perform basic skills and use the knowledge they have learned to analyze and solve practical problems through practical activities.
	Course difficulty level (A11)	The difficulty of the course refers to the depth and breadth of the course content required, as well as the setting of the evaluation criteria and test score lines.
Manage	Evaluation mechanism (A12)	The evaluation mechanism is the sum of various relationships between the evaluation subjects.
	Teaching supervision and management (A13)	Teaching supervision and management refers to the supervision and management of the teaching process and teaching quality.
	Organizational guarantee (A14)	The school’s guarantees in resource investment and institutional support provide material and policy foundation for improving participation.

### Bootstrapping resampling and weight assignment for ridge regression

To address the specific methodological challenges associated with the small expert panel used in this Delphi-based study, the combined application of Bootstrapping resampling and Ridge regression was adopted. This integration was designed to accomplish two principal objectives: first, to simulate a larger, more robust distribution of expert judgments from the limited sample of 12 experts (via Bootstrapping); and second, to derive stable, non-collinear expert weights from their potentially correlated scoring patterns (via Ridge Regression). Together, these techniques enhance the statistical reliability, objectivity, and stability of the expert opinion aggregation process, thereby providing a more rigorous foundation for the subsequent dynamic analysis.

Bootstrapping resampling is a statistical method that generates multiple subsample sets by drawing repeated random samples with replacement from the original dataset, which is particularly suitable for small sample data scenarios. In the Delphi method, Bootstrapping enhances the robustness and objectivity of weight assignment by generating multiple expert subsample sets and calculating the weight matrix for each subsample set.

First, by repeatedly resampling from the original set of expert ratings with replacement, the method effectively simulates a larger, more varied distribution of possible expert responses. This allows us to estimate the variability and stability of the derived weights—should a different subset of experts have been consulted—without the need to collect additional data. Consequently, the aggregated results become less sensitive to the specific composition of our panel, enhancing the robustness of our findings. Second, because the bootstrapped subsamples are generated randomly and each expert’s influence is evaluated across thousands of simulated samples, the final weight for each expert is not based on a single subjective judgment or a simple arithmetic average, but on a statistically aggregated outcome across many possible panel configurations. This process reduces the potential bias that could arise from over-reliance on any single expert’s ratings and provides a more objective, data-driven basis for integrating expert judgments into the final direct-influence matrix.

Ridge regression, as a linear regression model with regular terms, is particularly suitable for dealing with small-sample data scenarios in which there is a problem of multicollinearity among the independent variables. In this study, ridge regression was designed to use the scoring matrix of each of the 12 experts on the degree of influence between the factors as the independent variable, and the weight matrix of each expert as the dependent variable, and the weights were obtained through the regression coefficients. This design solves the problem of multicollinearity in the weight assignment process of the traditional Delphi method, avoids the risk of overfitting, and improves the stability of the weights.

When experts evaluate interrelated factors, their ratings are frequently correlated, leading to multicollinearity among the independent variables (the expert scoring matrices). This collinearity can cause high variance in the estimated weights—small changes in the input data might lead to large, unstable shifts in the assigned weights, compromising the reliability of the aggregated group matrix.

Ridge regression directly mitigates this issue by introducing an L2 regularization term (*λ*∑βⱼ^2^) to the loss function. This penalty term shrinks the regression coefficients toward zero, effectively damping the excessive influence of any single expert’s correlated rating pattern. By constraining the magnitude of the coefficients, the model becomes less sensitive to the multicollinearity present in the small-sample expert data. A model overfits when it captures idiosyncratic variations in the small sample rather than the underlying the true consensus trend. The regularization in ridge regression reduces model complexity, preventing it from fitting these random fluctuations in the training data and thus improving its generalizability.

Consequently, the final expert weights estimated via ridge regression are more stable and robust. This approach ensures that the integration of expert judgments is both statistically sound and resilient to the inherent correlations in subjective ratings.

The loss function of ridge regression can be expressed as:


$$J(\beta )=\sum_{i=1}^{n}{\left(y_{i}-\hat{y_{i}}\right)}^{2}+\lambda \sum_{j=1}^{p}\beta_{j}^{2}$$

$y_{i}$ is the target value for the 𝑖 th observation, which is the equalization target for the expert weights in this study, and is set to be an all-1 vector.

$\hat{y_{i}}=X_{i}\beta$ is the predicted value of the model, where $X_{i}$ is the eigenvector after flattening the scoring matrix of the experts.

$\beta_{j}$ is the regression coefficient, which indicates the contribution of each influencing factor to the expert weights.

λ is the ridge parameter, the more significant the coefficient contraction, the less complex the model.

In this study, the logarithmic scale search strategy of K-fold cross-validation is used to systematically evaluate the model generalization ability under differentλ values, and finally select the parameter values that minimize the mean square error (MSE) of prediction, whose mathematical expressions are as follows:


$$\mathrm{MSE}=\frac{1}{m}\sum_{i=1}^{m}{\left(y_{i}-\hat{y_{i}}\right)}^{2}$$

wherem is the number of validation set samples. In this study, the bootstrapping process employed simple random sampling to select 5 out of the 12 experts’ scores each time, forming a sub-sample set. By repeating this process 7,920 times, 7,920 influence matrix samples were generated. Based on this subsample set, more accurate and appropriate ridge parameters can be obtained.

To substantiate the reliability of the direct-influence matrix W, an extensive bootstrapping validation comprising 7,920 iterations was applied to the 14 matrices obtained from the expert panel.

The validation confirmed the robustness of matrix W across three critical dimensions. First, regarding precision, the weights assigned to each expert were characterized by narrow 95% confidence intervals, with an average width of 0.200. This indicates a well-defined and stable contribution structure among the experts. Second, a sensitivity analysis demonstrated the matrix’s high stability against resampling variability. This was evidenced by a strong mean correlation of 0.966 and a concurrent low root mean square error of 0.318 observed between the final integrated matrix and a random subset of 100 bootstrap candidate matrices. Third, convergence diagnostics verified that the key statistical estimates stabilized well before completing the full set of 7,920 iterations, confirming the adequacy of the resampling scope.

Consequently, the resulting matrix W—which itself shows a high correlation of 0.961 with the underlying expert consensus structure—serves as a statistically robust foundation. This ensures that the subsequent dynamic MICMAC trajectories and derived patterns reflect genuine systemic properties rather than artifacts of sampling variability.

**Figure 2:**
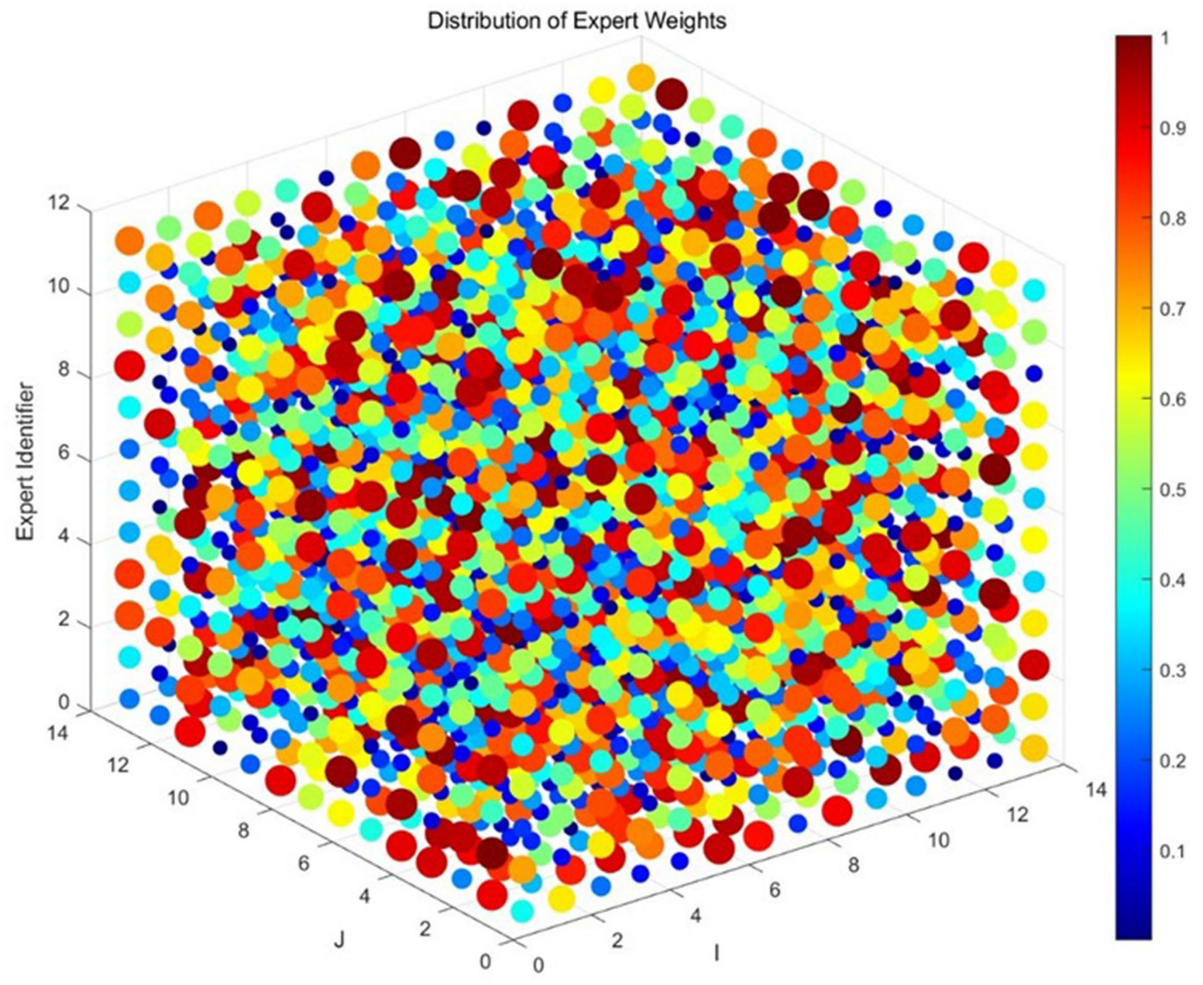
Distribution of expert weights.

The final weight distribution of the 12 experts is shown in Figure 2.

After obtaining the final weighting matrix $M_{i}(i=1,2,\ldots ,12),$ a weighted average of the original 12 experts’ scoring matrix $N_{i}(i=1,2,\ldots ,12)$ is performed. The final direct impact matrix W can be computed by arrive at Table 3.


$$W=\sum_{i=1}^{12}M_{i}\times N_{i}$$

**Table 3:** Final direct impact matrix W.

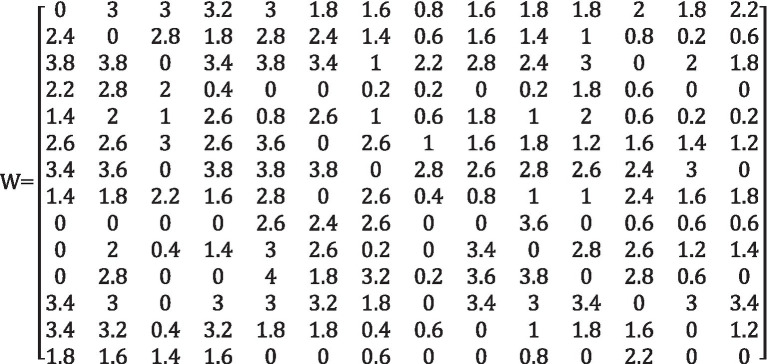

### Time-varying influence network model construction

The Time-Varying Influence Network (TVIN) model is a dynamic model that captures the influence relationships between variables in a system over time. Compared with traditional static network models, TVIN models can more accurately simulate system dynamics and improve prediction capability and decision support.

In this study, the TVIN model is able to dynamically track the evolution of the influence matrix between parent nodes through dynamic system theory and difference equations; the state feedback mechanism in it avoids the numerical overflow problem.

Within the dynamic analysis, a modeled time step functions as a proxy for a distinct phase in school development. This phase corresponds to a coherent period within an instructional cycle—such as an academic year or a multi-year program—characterized by a shift in the system’s state. The progression from initial implementation of educational practices to their subsequent institutionalization defines these phases. This cycle typically progresses from a stage of initial implementation and adaptation, where new policies, curricula, or instructional approaches are introduced, to a subsequent stage of routinization and systemic integration, where these elements become stabilized within the educational environment. Each modeled time step, therefore, corresponds to a period in which the school system undergoes measurable shifts in the relationships and influences among key factors. Thus, the model’s iterative evolution is explicitly designed to simulate the non-linear transitions that occur as a school setting matures, ensuring the mathematical dynamics are directly interpretable within a real-world developmental trajectory.

The model assumes that the range of values of the state variable X is restricted to be between 0 and 100, value of 100 indicates that the performance is optimal. In this paper, the initial state of each node of the system is set to 1, and the process from the initial state to the system converging to the steady state is investigated. The rationale for setting the initial state of all system nodes to a uniform value of 1 is directly derived from the prior expert evaluation phase. As detailed in the Delphi procedure for assessing inter-factor influences, experts were explicitly instructed to rate the direct influences between factors under the premise that all factors had been standardized to a common scale of impact on student engagement. It represents a neutral, non-zero baseline from which the dynamic interplay of influences, as captured by the time-varying matrix V(t), can evolve. This approach allows the model simulation to trace the system’s development from a state of equal latent potential toward its emergent steady state, effectively capturing the differential amplification of influences over time that arises purely from the network’s relational structure, rather than from arbitrary initial advantages among factors.

Based on these assumptions, the state transfer equations of the TVIN model are designed as:


$$X_{i}(t+1)=X_{i}(t)+\alpha \cdot (1-\frac{X_{i}(t)}{100})\cdot \sum_{j=1}^{14}W_{\mathrm{ij}}\cdot X_{j}(t)$$

X is the state matrix of each node, $X_{i}(t)$ is the state value of node i at point in time t,W is the direct influence matrix, and $W_{\mathrm{ij}}$ represents the influence of node j on node i.

α is the coefficient that controls the update rate.

To ensure the model reliably simulates the system’s evolution from its initial state to a steady state and to justify the selection of the update coefficient, explicit quantitative criteria were established to determine convergence. Theoretically, the system reaches a steady state when the state change for all nodes approaches zero.

The fixed value of *α* = 0.0025 ensures the system’s dynamic evolution is fully captured while maintaining numerical stability. This parameter balances responsiveness to transient fluctuations and long-term convergence, allowing the model to trace the entire developmental trajectory of node interactions from initial activation to steady-state stabilization.

At time point t, the direct influence matrix between nodes is denoted as V(t), and at time point t, the strength of direct influence of node j on node i is:


$$V_{\mathrm{ij}}(t)=W_{\mathrm{ij}}\cdot X_{j}(t)$$

In each iteration, the direct influence matrix between nodes is updated by updating the state matrix of each node X.

### Dynamic MICMAC analysis

MICMAC (Matrix of Influence and Dependence Analysis) is a methodology used to assess and analyze the interactions between factors within a system. It discerns which factors are drivers and which are dependents by constructing a direct influence matrix and performing a series of mathematical processes on it. In this paper, MICMAC analysis is performed on the direct influence matrix V(t) at each point in time to capture the changes in the attributes of the influencing factors as the direct influence matrix changes over time.

The total impact matrix T(t) contains both direct and indirect impacts. The indirect impact matrix is calculated by the following formula:


T(t)=V(t)+I(t)

$$I(t)=V{\left(t\right)}^{2}+V{\left(t\right)}^{3}+V{\left(t\right)}^{4}+\cdots =\sum_{k=2}^{\infty }V{\left(t\right)}^{k}$$

Driver Power indicates the overall influence of a factor on other factors. For the factor i its Driver Power can be obtained by calculating the sum of all the elements in the row i.


$$DP_{i}(t)=\sum_{j=1}^{14}T_{\mathrm{ij}}(t)$$

Dependency: indicates the degree to which a factor is influenced by other factors. For the factor i its dependency can be obtained by calculating the sum of all the elements in the column i:


$$DE_{i}(t)=\sum_{j=1}^{14}T_{\mathrm{ji}}(t)$$

While the traditional static MICMAC method only analyzes once based on the initial weight matrix, the dynamic MICMAC method in this study is capable of continuously updating the weight matrix over time to capture the time-varying characteristics of factor attributes. It is able to fully explore the changes in the driving and dependent capabilities of each node at different time steps.

Operationally, convergence was defined by two stringent criteria evaluated over a final observation window of M timesteps (here, M=20). The system was considered to have converged if, during this window the maximum absolute change across all nodes fell below a threshold:


$$\max_{i,t\in [T-M+1,T]}|\mathrm{\Delta D}P_{i}(t)|<\delta_{\mathrm{abs}},\text{with}\;\delta_{\mathrm{abs}}=0.05$$

$$\max_{i,t\in [T-M+1,T]}|\mathrm{\Delta D}E_{i}(t)|<\delta_{\mathrm{abs}},\text{with}\;\delta_{\mathrm{abs}}=0.05$$

The update coefficient was set to α=0.0025 following preliminary sensitivity analyses.

The simulation results confirm that with α=0.0025, the system satisfies both convergence criteria after approximately t>170 timesteps. Specifically, throughout the final 20-timestep window, the maximum absolute state change remained below 0.05, and the mean relative rate of change was consistently under 0.1%. This numerical evidence demonstrates that the chosen α value robustly guides the system from its initial state to a well-defined and stable equilibrium, thereby providing a reliable foundation for analyzing its dynamic evolution patterns.

## Results

**Figure 3:**
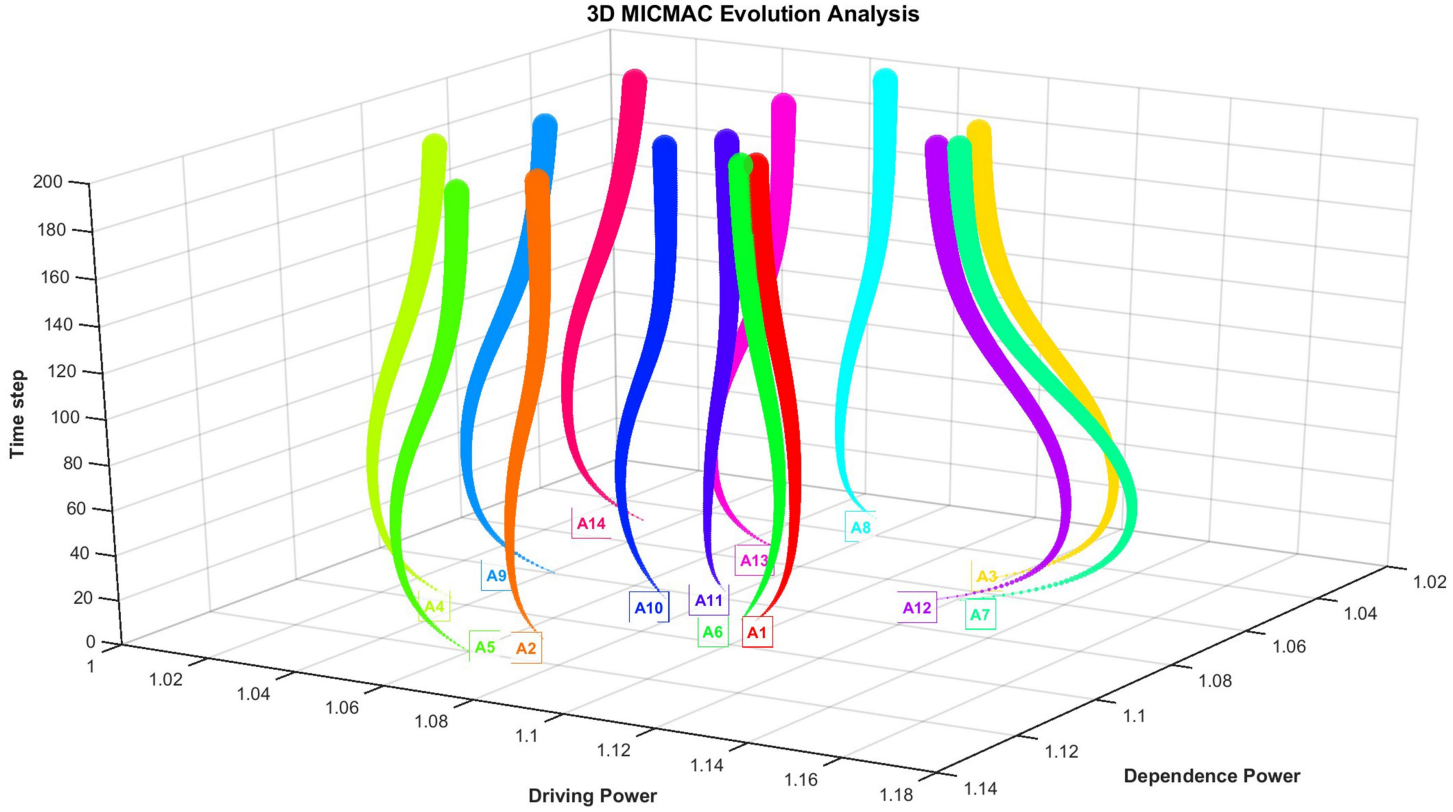
Three-dimensional MICMAC analysis over time.

The results of the MICMAC analysis of each node over time are shown in Figure 3, which demonstrates the changes in the driving and dependent capabilities of each node at different time steps. Each node is represented by a different color and the data points from each time step are connected to form a trajectory. The later the time, the larger the data points. Figure 3 demonstrates the trend of the state values of each of the 14 nodes over time.

As shown in Figure 3, most factors exhibit significant fluctuations, peaking at a time step of 50. Initially, they undergo a sharp change; after reaching the peak, the trend reverses and the rate of change gradually slows down.

A11 shows little fluctuation throughout the period, while A1 and A6 exhibit relatively smaller variations.

**Figure 4:**
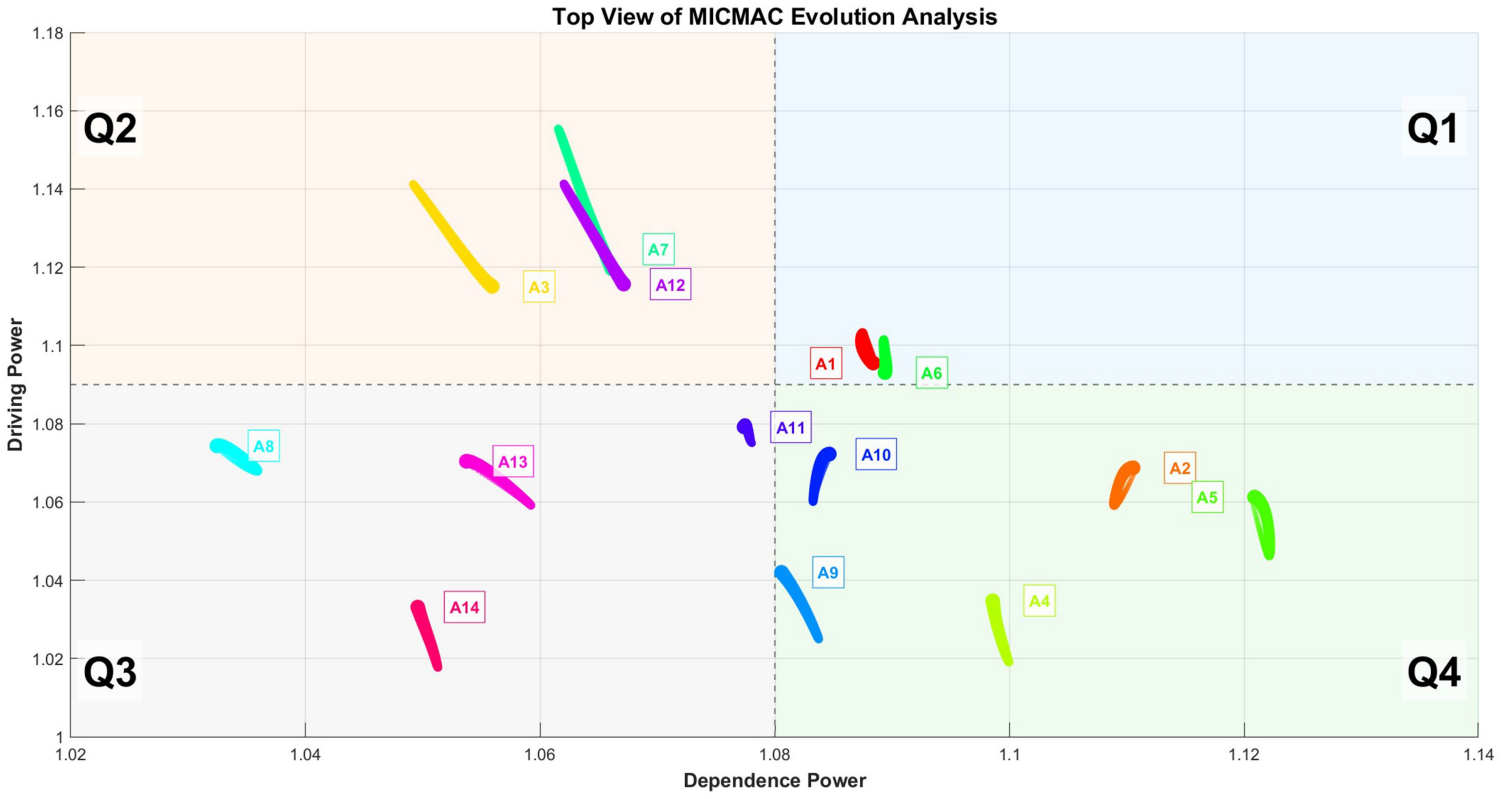
Top view of the trajectories of the 14 factors over time.

Figure 4 is a top view of the trajectory of the MICMAC analysis results over time. Correspondingly, in the top-view trajectory plot, thicker line segments represent more recent time steps.

The top view of the MICMAC evolution analysis illustrates the dynamic trajectories of 14 system factors (A1–A14) across multiple time steps, mapped in a two-dimensional space defined by Dependence Power (x-axis) and Driving Power (y-axis). The chart is divided into four quadrants—Q1 (Dependent), Q2 (Linkage), Q3 (Autonomous), and Q4 (Driving)—to categorize the strategic roles of each factor based on their influence and dependency characteristics. Factors located in Q1 exhibit high dependence and high driving power, indicating they are influential but highly influenced by others; those in Q2 possess low dependence and high driving power, suggesting they are key leverage points with strong impact and minimal external control, making them critical for intervention strategies. Factors in Q3 show low dependence and low driving power, implying autonomy with limited influence, while Q4 factors have high dependence and low driving power, meaning they are strongly driven by other elements but exert little influence themselves. The color-coded trajectories represent individual nodes, with colors corresponding to node numbers as shown in the legend, and the thickness and opacity of the lines indicate temporal progression, where later time steps are more prominent.

Over time, the driving power of most nodes tends to converge toward the median value, particularly evident among factors initially located in the fourth quadrant (Driving zone), such as A2, A4, A5, A9, and A10.

Notably, factors A1 and A6 remain consistently positioned within the first quadrant (Dependent zone) throughout the entire evolutionary process, exhibiting minimal fluctuation in both driving and dependence powers. This stability indicates that these factors maintain a persistent role as highly interconnected elements—simultaneously exerting and receiving significant influence within the system. Their limited movement suggests a state of relative equilibrium, where their influence levels do not undergo substantial temporal shifts, reflecting either intrinsic robustness or integration into stable feedback loops. Unlike other factors that display pronounced dynamic transitions, the consistent trajectories of A1 and A6 highlight their role as structurally stable components within the evolving system, potentially serving as anchors that contribute to systemic coherence despite ongoing changes in other parts of the network. Their dual capacity to consistently exert and receive significant influence suggests they are embedded in core feedback loops that regulate system behavior. This intrinsic robustness makes them non-negotiable elements for systemic coherence; they provide a stable reference frame that dampens excessive volatility and maintains functional continuity amidst the dynamic shifts exhibited by other factors. This convergence suggests a dynamic equalization process within the system, where initially dominant drivers become more balanced in their influence, potentially reflecting stabilization, increased interdependence, or the diffusion of control across multiple factors. The observed movement highlights a shift from a centralized influence structure toward a more distributed and integrated system behavior, with the original strong drivers gradually aligning with the system’s average driving capacity.

Notably, factors A3, A7, and A12—located in the first quadrant (Dependent zone)—exhibit a distinct temporal pattern in their driving power: a significant initial increase followed by a gradual decline. This trend indicates that these factors initially gained strong influence within the system, likely acting as key drivers during the early stages of evolution. However, as the system progressed, their relative driving capacity diminished, suggesting a shift in systemic dynamics where their influence became less dominant. The behavior of A3, A7, and A12 underscores the dynamic and non-linear nature of factor interactions, highlighting that high influence in early phases does not necessarily imply sustained dominance over time.

In contrast, A1, A8, A11 and A14 remain in Q3 throughout, indicating persistent autonomy and limited systemic impact.

## Discussion

Figure 4 presents a dynamic top view of the MICMAC evolution analysis, revealing complex temporal patterns in the interplay between driving and dependence powers of 14 system factors (A1–A14). The results demonstrate that the system undergoes a non-linear transformation over time, characterized by three distinct behavioral patterns:

(1) Convergence toward equilibrium, as most nodes—especially initial strong drivers in Q4 such as Teaching methods (A2), Learning Methods (A4), Student course adaptation (A5), Course interactive design (A9), and Course practice session (A10)—gradually reduce their driving power toward the median, indicating a shift from centralized to distributed influence. This evolution can be interpreted within the practical context of physical education (PE) pedagogy. For example, a novel teaching method (A2) may initially exert strong influence as students and teachers adapt to it; however, as it becomes embedded in routine practice, its relative impact stabilizes. Similarly, a structured course practice session (A10) might powerfully engage students when first introduced, but its driving effect diminishes as other elements—such as peer interaction or increased student autonomy—become integrated into the learning environment. Essentially, the system undergoes a process of dynamic normalization: factors that are salient or innovative in early stages become absorbed into a more balanced network of influences as the educational setting matures. This finding underscores that PE engagement is not sustained by a few dominant factors in the long term, but by the increasingly balanced and interdependent contributions of multiple elements. In practical terms, this suggests that while introducing innovative methods or interactive designs can boost engagement initially, long-term strategies should focus on cultivating a coherent and well-integrated pedagogical ecosystem rather than over-relying on any single intervention.
(2) Temporary dominance, where factors like Teaching work attitude (A3), Personal preferences (A7), and Evaluation mechanism (A12) experience a surge in driving power followed by a decline, reflecting transient leadership roles shaped by early-stage dynamics and subsequent feedback adjustments. This pattern suggests that their influence peaks during formative or transitional periods, serving as catalysts that initiate systemic change. As the school environment matures and structural elements—such as institutional support and curricular coherence—become more established, the system’s reliance on these transient drivers decreases. Notably, lower levels of these factors may suffice to sustain student engagement as the overall context evolves, indicating a shift in the system’s dependency structure. In practice, these findings highlight the importance of strategically timing interventions targeting A3, A7, and A12 early in the developmental cycle to leverage their catalytic potential, while later efforts should focus on reinforcing stable, long-term drivers to maintain engagement momentum.
(3) Structural stability, exemplified by Professional teaching ability (A1) and Student course identification (A6), which remain firmly in Q1 with minimal fluctuation, acting as consistently influential and highly interconnected hubs throughout the evolution. For practice, this implies that long-term strategies must prioritize reinforcing these anchor factors—for instance, through sustained teacher development and initiatives to foster curriculum relevance—as they are essential for maintaining systemic resilience and integrating the effects of other targeted interventions.

In contrast, Interesting course (A8), Course difficulty level (A11), and Organizational guarantee (A14) persist in Q3 (Autonomous zone), showing low influence and low dependency, suggesting marginal roles in system dynamics. These patterns collectively illustrate a system evolving toward greater integration and balance, where initial asymmetries in influence are gradually mitigated through increasing interdependence. This dynamic highlights a critical practical insight: sustainable student engagement in physical education relies not on transient or isolated factors like momentary enjoyment or resource provision alone, but is fundamentally anchored by the stable, reciprocal relationship between teacher expertise and deep student buy-in, around which other elements can be effectively orchestrated.

Our findings align with the theoretical propositions of Self-Determination Theory, particularly in the identification of factors that exhibit structural stability or transient dominance. For instance, factors such as Professional teaching ability (A1) and Student course identification (A6) consistently function as highly interdependent elements, reflecting their role in supporting students’ needs for competence and relatedness—key components of SDT. Similarly, the transient dominance of factors like Teaching work attitude (A3) and Personal preferences (A7) may correspond to autonomy-supportive or autonomy-thwarting behaviors that vary in salience over time, as highlighted in recent SDT-based classifications of teacher behaviors. The observed convergence of driving power toward equilibrium further suggests a system moving toward balanced need satisfaction, reinforcing SDT’s emphasis on the dynamic and context-sensitive nature of motivational processes.

### Limitations

Although this study tried its best to invite experts from different backgrounds to participate in the Delphi method and attempted to analyze the problem from multiple perspectives, the selected sample size is relatively small and mainly focused on specific regions, which may affect the generalizability and representativeness of the results.

The reliance on a panel of 12 experts constitutes a further constraint. While bootstrapping was employed to mitigate issues related to small sample size, the limited initial expert pool may still affect the robustness of the identified factor structure and influence relationships. Additionally, as shown in Table 1, the panel consisted predominantly of senior scholars and experienced teachers. Although this composition provides valuable expertise, it may not fully incorporate the perspectives of early-career educators or students themselves, whose views directly shape engagement dynamics. This potential bias in perspective could influence the weighting and interpretation of factors.

Although the TVIN model has improved in capturing dynamic changes compared with the traditional model, in practical applications, some characteristics of complex systems are difficult to be fully predicted by the model, and more empirical studies are still needed to verify its effectiveness. Additionally, it should be acknowledged that the TVIN model, while designed to capture temporal dynamics, remains a computational simulation based on expert judgments elicited at a single point in time. Its “dynamic” evolution is projected from an initial consensus, rather than being empirically derived from or validated against longitudinal measurements of actual student engagement over a school development cycle. This represents a primary limitation in asserting that the modeled trajectories fully mirror real-world educational evolution. Although the model provides a valuable theoretical framework for understanding potential systemic interactions and phase transitions, its predictive accuracy and external validity require confirmation through future studies that collect and utilize longitudinal engagement data alongside the expert-informed structural parameters.

## Conclusion

The evolutionary trajectory analysis reveals that while certain factors initially dominate the system’s dynamics, their influence tends to diminish or stabilize over time, leading to a more balanced and interconnected structure. The system transitions from an early phase of heterogeneous influence distribution to a later state of relative equilibrium, driven by feedback mechanisms and growing mutual dependencies. Key factors such as Professional teaching ability (A1) and Student course identification (A6) emerge as stable core elements, while others like Teaching work attitude (A3), Personal preferences (A7), and Evaluation mechanism (A12) play pivotal but time-limited roles. Meanwhile, factors in Q3 remain peripheral, contributing little to systemic change.

Based on these findings, it is evident that the timing and targeting of interventions play a critical role in shaping system outcomes, which leads to several actionable recommendations for practice. Future interventions in physical education should consider adopting evidence-based teacher motivational behaviors—such as providing rationales, offering meaningful choices, and fostering relatedness to create more adaptive and responsive learning environments.

Leverage transient drivers strategically: Factors such as Teaching work attitude (A3), Personal preferences (A7), and Evaluation mechanism (A12) exhibit strong early influence. Interventions should be implemented early in the system evolution to maximize their effectiveness.

Strengthen stable hubs for resilience: Given their persistent role, Professional teaching ability (A1) and Student course identification (A6) should be prioritized for reinforcement and monitoring, as they contribute significantly to system coherence and may serve as anchors during periods of change.

Leverage transient drivers strategically: Factors such as Teaching work attitude, Personal preferences, and the Evaluation mechanism exhibit strong influence during early stages. To maximize their impact, targeted interventions should be implemented at the outset of a course or academic year. For Teaching work attitude, effective early-stage intervention centers on the educator’s deliberate self-reflection and behavioral adjustment. PE teachers can actively cultivate a positive teaching disposition by routinely examining their own instructional engagement. This inward-focused practice, aimed at aligning observable teaching behaviors with a supportive and enthusiastic professional mindset, can establish a strong motivational tone from the very beginning of the course. For the Evaluation mechanism, its effectiveness hinges on clarity and consistency; therefore, teachers should explicitly communicate assessment criteria, introduce formative feedback tools, and establish routine check-ins within the first few weeks to build student trust and understanding of performance expectations.

Strengthen stable hubs for resilience: The foundational factors of Professional teaching ability and Student course identification require ongoing and deliberate support. Enhancing Professional teaching ability involves a commitment to continuous professional development. This can be operationalized through regular participation in pedagogical training workshops focused on physical education, engagement in collaborative lesson planning with colleagues, and dedicated time for reviewing current research in sports pedagogy to inform and refine teaching practices. Fostering Student course identification is a sustained process of meaning-making. Teachers can achieve this by consistently articulating the connections between course activities, personal health goals, and broader life skills during lessons, and by creating opportunities for students to reflect on and document their own progress and achievements throughout the course.

Anticipate equalization of influence: As initially dominant drivers lose relative influence, the driving power of most nodes tends to converge toward the median value, long-term strategies should avoid over-reliance on any single factor and instead promote distributed governance or adaptive management frameworks.

## Data Availability

The original contributions presented in the study are included in the article/supplementary material, further inquiries can be directed to the corresponding author.

## References

[ref1] Ahmadi A. Noetel M. Parker P. Ryan R. M. Ntoumanis N. Reeve J. . (2023). A classification system for teachers’ motivational behaviors recommended in self-determination theory interventions. J. Educ. Psychol. 115, 1158–1176. doi: 10.1037/edu0000783

[ref2] Babic M. J. Morgan P. J. Plotnikoff R. C. Lubans D. R. Eather N. Batterham M. (2014). Physical activity and physical self-concept in youth: systematic review and meta-analysis. Sports Med. 44, 1589–1601. doi: 10.1007/s40279-014-0229-6 25053012

[ref3] Caspersen C. J. Powell K. E. Christenson G. M. (1985). Physical activity, exercise, and physical fitness: definitions and distinctions for health-related research. Public Health Rep. 100, 126–131. 3920711 PMC1424733

[ref4] Chai Y. Wang Y. Zhu L. (2021). Information sources estimation in time-varying networks. IEEE Trans. Inf. Forensics Secur. 16, 2621–2636. doi: 10.1109/TIFS.2021.3054745

[ref5] Clemen R. T. Winkler R. L. (1999). Combining probability distributions from experts in risk analysis. Risk Anal. 19, 187–203. 10.1111/0272-4332.202015 10859775

[ref6] Fairclough S. J. Stratton G. (2006). Effects of a physical education intervention to improve student activity levels. Phys. Educ. Sport Pedagog. 11, 29–44. doi: 10.1080/17408980500469137

[ref7] Haider S. Levis A. H. (2008). Modeling time-varying uncertain situations using dynamic influence nets. Int. J. Approx. Reason. 49, 488–502. doi: 10.1016/j.ijar.2008.05.001

[ref8] Jetter A. Schweinfort W. (2011). Building scenarios with fuzzy cognitive maps: an exploratory study of solar energy. Futures 43, 52–66. doi: 10.1016/j.futures.2010.10.005

[ref9] Jiang X. Lu K. Xia B. Skitmore M. Long R. (2019). Identifying significant risks and analyzing risk relationship for construction PPP projects in China using integrated FISM-MICMAC approach. Sustainability 11:5206. doi: 10.3390/su11195206

[ref10] Keegan R. J. Barnett L. M. Dudley D. Telford R. D. Lubans D. R. Bryant A. S. . (2019). Defining physical literacy for application in early childhood contexts: an expert consensus study. Front. Psychol. 10:2702. doi: 10.3389/fpsyg.2019.02702, 31849792 PMC6901422

[ref11] Lonsdale C. Rosenkranz R. R. Peralta L. R. Bennie A. Fahey P. Lubans D. R. (2013). A systematic review and meta-analysis of interventions designed to increase moderate-to-vigorous physical activity in school physical education lessons. Prev. Med. 56, 152–161. doi: 10.1016/j.ypmed.2012.11.021, 23246641

[ref12] McDonald G. C. (2009). Ridge regression. Wiley Interdiscip. Rev. Comput. Stat. 1, 93–100. doi: 10.1002/wics.15

[ref13] Nevitt J. Hancock G. R. (2001). Performance of bootstrapping approaches to model test statistics and parameter standard error estimation in structural equation modeling. Struct. Equ. Model. 8, 353–377. doi: 10.1207/S15328007SEM0803_2

[ref14] Reeve J. Cheon S. H. (2021). Autonomy-supportive teaching: its mallea bility, benefits, and potential to improve educational practice. Educ. Psychol. 56, 54–77. doi: 10.1080/00461520.2020.1862657

[ref15] Rowe G. Wright G. (2011). The Delphi technique: past, present, and future prospects—introduction to the special issue. Technol. Forecast. Soc. Change 78, 1487–1490. doi: 10.1016/j.techfore.2011.07.002

[ref16] Ryan R. M. Deci E. L. (2017). Self-determination theory: Basic psycho logical needs in motivation, development, and wellness. Guilford Press. Available online at: https://play.google.com/store/books/details?id=GF0ODQAAQBAJ

[ref17] Spittle M. Jackson K. Casey M. (2009). Applying self-determination theory to understand the motivation for becoming a physical education teacher. Teach. Teach. Educ. 25, 190–197. doi: 10.1016/j.tate.2008.07.002

[ref18] Xiang P. Agbuga B. Liu W. Solmon M. A. (2017). Student motivation in physical education: a self-determination theory perspective. J. Teach. Phys. Educ. 36, 289–297. doi: 10.1123/jtpe.2017-0056

